# Effective and Evidence-Based: How the 4Ms Age-Friendly Framework Can Result in Improved Care in a Rural Primary Care Clinic

**DOI:** 10.1093/geroni/igaa057.446

**Published:** 2020-12-16

**Authors:** Leah Tobey, Robin McAtee

**Affiliations:** 1 University of Arkansas for Medical Sciences, Little Rock, Arkansas, United States; 2 UAMS, Little Rock, Arkansas, United States

## Abstract

As healthcare providers struggle to reframe aging, framing Age-Friendly care is also occurring. The Arkansas Geriatric Education Collaborative (AGEC) is a HRSA Geriatric Workforce Enhancement Program with an objective to improve clinical health outcomes of older adults (OA) in primary care settings. As a member of the 2020 Institute for Healthcare Improvement (IHI) Age-Friendly cohort, the AGEC has partnered with ARcare, an AR federally qualified healthcare clinic network, to implement the 4Ms in 4 rural clinics over 3 years. AGEC’s first goal of working with rural primary care clinics is to improve their knowledge of best practices of caring for OA. This was started by providing Geriatric Interdisciplinary Team Training to clinic staff, obtaining baseline data of common health related indicators for OA and starting regular geriatric focused training. Training on the 4Ms (Matters, Medication, Mentation Mobility) framework was next and completed followed by planning and implementation. The process was well received and results are promising. Year 1 data in one clinic show incremental improvements over baseline data in several areas including assessing Mobility with fall screens which has improved over 50% in one year and annual wellness visits (where all 4Ms are reviewed) have increased 30%. However, several areas of opportunities for improvement have also been noted and turned into quality improvement projects (QI). This includes an opportunity to improve depression screens for the clinic’s Mentation measure, which dropped almost 30% in one year. QI projects are ongoing to improve each of the elements of becoming age-friendly.

